# Supplementary material for: InterCellar enables interactive analysis and exploration of cell−cell communication in single-cell transcriptomic data
Source: Commun Biol. 2022 Jan 11;5:21. doi: 10.1038/s42003-021-02986-2 (PMC8752611; doi:10.1038/s42003-021-02986-2)
Supplement: Supplementary file 2 — Supplementary Information [file 42003_2021_2986_MOESM2_ESM.pdf]

InterCellar enables interactive analysis and exploration of cell-cell communication in single-cell transcriptomic data

Marta Interlandi[1,2]*, Kornelius Kerl[2] and Martin Dugas[1,3]

[1] Institute of Medical Informatics, University of Münster, Münster, Germany
[2] Department of Pediatric Hematology and Oncology, University Children's Hospital Münster, Münster, Germany
[3] Institute of Medical Informatics, Heidelberg University Hospital, Heidelberg, Germany

*Correspondence: marta.interlandi@uni-muenster.de

Supplementary Information

Supplementary Note 1: Input data from tools supported by InterCellar

In order to increase the general usability of InterCellar, four published methods allowing the prediction of cell-cell communication from scRNA-seq data are automatically supported as input tools to InterCellar[1–4]. This allows straightforward interoperability between InterCellar and the chosen tool, with no further data preparation required. Here we describe in detail the

characteristics of the cell-cell interactions dataset (CCI data) that InterCellar expects as input, depending on the supported tool chosen.

CellChat

Users running the CellChat[1] R package to obtain predicted cell-cell interactions should save the dataframe generated by the CellChat function subsetCommunication as a .csv file (or .xlsx/.tsv). This can be accomplished by following the steps suggested in the CellChat tutorial (https://htmlpreview.github.io/?https://github.com/sqjin/CellChat/blob/master/tutorial/CellChat-vignette.html). We recommend the R function write.csv (with parameter quote = FALSE) to save the dataframe into a .csv file that can be loaded by InterCellar. A snippet of the dataframe generated on the melanoma data can be found as an example in Supplementary Table 2. InterCellar automatically parses the dataframe and generates preprocessed CCI data by restructuring the information as follow:

- Int_pair: from column “interaction_name_2”, substituting “-” with “&”;
- geneA, geneB: retrieved from “interaction_name_2”, split by “-”;
- typeA = L (ligand), typeB = R (receptor);
- clustA = “source”, clustB = “target”;
- Score = “prob”;
- P_value = “pval”;
- Int.type = autocrine (if clustA=clustB) or paracrine (otherwise);
- Pathway_cellchat = “pathway_name”;
- Annotation_cellchat = “annotation”;
- Evidence_cellchat = “evidence”.

CellPhoneDB v2

Users adopting CellPhoneDB v2[2] (CPDB) as an inference method have the option of running a statistical analysis that computes p-values of significance for each interaction. CPDB tutorials can be found at https://github.com/Teichlab/cellphonedb. When the statistical analysis has been run, the user will find four output .txt files corresponding to “deconvoluted”, “means”, “pvalues”, and “significant_means”. In this case, InterCellar relies on the “significant_means” and “pvalues” files, otherwise, on the file “means”. Thus, as input to InterCellar, the user must specify the output folder generated by CPDB. An example of the “significant_means” file can be seen in Supplementary Table 3. In order to build the

preprocessed CCI data, InterCellar relies on CPDB specific annotation that is either included in the supplied files (e.g., “significant_means”) or retrieved from CPDB documentation, namely the gene_input and complex_input tables (https://www.cellphonedb.org/documentation). Moreover, as CPDB considers also multi sub-units complexes, InterCellar will retain this information and integrate it with simple interaction-pairs. Information is mapped as follows:

- Int_pair: gene_a & gene_b, or name of complex;
- geneA = gene_a or list of gene symbols separated by “,” for complexes, retrieved from complex_input table;
- geneB = gene_b or list of gene symbols separated by “,” for complexes, retrieved from complex_input table;
- typeA, typeB: depending on “receptor_a” and “receptor_b” columns;
- clustA, clustB: from the col.names of the table, split by “|”;
- Score = “mean_value”;
- P-value: retrieved from the “pvalues” file, when present;
- Annotation_strategy = “annotation_strategy”;
- Int.type = autocrine (if clustA=clustB) or paracrine (otherwise);

Due to the fact that CPDB does not order int-pairs as L-R pairs, InterCellar will reorder all pairs (and respective cluster-pairs) to comply with an L-R arrangement. Moreover, we manually updated the annotation to L or R of certain genes that were incorrectly annotated by CPDB (https://github.com/martaint/InterCellar-reproducibility/blob/main/preprocessing/checkLL_RR.R). Importantly, int-pairs collected by CPDB are composed of L-R, L-L, and R-R pairs, thus providing an explanation as to why the labeling of undirected interactions by InterCellar (L-L and R-R).

ICELLNET

ICELLNET[4] requires the user to select (1) a cell cluster of interest (i.e., central cell) and (2) a direction of communication (“in” for incoming, “out” for outgoing), from which to compute the cell-cell interactions. This information must be provided by the user in InterCellar’s upload section, along with a dataframe generated by ICELLNET’s function icellnet.score (see the tutorial at https://github.com/soumelis-lab/ICELLNET/blob/master/Exemple2_scRNAseq.md). This dataframe should be saved as a .csv file with the R function write.csv (with parameter row.names = TRUE). An example of ICELLNET’s dataframe is shown in Supplementary Table 4. In this case, we consider as central cell malignant cells from the melanoma dataset,

and outgoing communication towards all other clusters. InterCellar parses the dataframe and generates preprocessed CCI data as follow:

If direction = “out”:

- Int_pair: row.names of the dataframe, substituting “ / “ with “&”;
- geneA, geneB: retrieved from row.names, split by “ / ”;
- typeA = L (ligand), typeB = R (receptor);
- clustA = name of the central cell, clustB = col.names taken from ICELLNET dataframe;
- Score = numerical entries of the dataframe;
- Int.type = autocrine (if clustA=clustB) or paracrine (otherwise).

When direction = “in”, clustA would be taken from col.names, while clustB = central cell.

SingleCellSignalR

Instructions on how to run SingleCellSignalR[3] (SCSR) to obtain predicted CCI data can be found at https://bioconductor.org/packages/release/bioc/vignettes/SingleCellSignalR/inst/doc/UsersGuide.html. InterCellar expects as input the folder called “cell-signaling” generated by running the SCSR function cell_signaling. This folder contains multiple .txt files for each cluster-pair considered. A snippet of an example file can be found in Supplementary Table 5. InterCellar parses each of these files and combines the information into a single preprocessed CCI data, as follow:

- Int_pair: from elements in first and second columns of SCSR files, separated by “&”;
- geneA: elements from first column, geneB: elements from second column;
- typeA = L (ligand), typeB = R (receptor);
- clustA = col.name of first column; clustB = col.name of second column;
- Score = “LRscore”;
- Int.type = autocrine (if clustA=clustB) or paracrine (otherwise). As SCSR defines as “autocrine|paracrine” interactions that are paracrine but are also found as autocrine, we rename these “paracrine” to be consistent with other tools;
- scSignalR_specific: TRUE for interactions labeled as “specific” by SCSR, when both ligand and receptor are significantly enriched in their respective clusters.

Supplementary Note 2: Filtering options provided in the gene-verse

InterCellar's gene-verse provides the user with filtering options that are specific to the input tool chosen. As a reminder, these filters have a global impact, subsetting the input data for further analyses. Here we present in detail which filters are provided, depending on the input tool.

CellChat

CellChat-specific filters regard the pathway annotation of int-pairs (column "pathway_cellchat") and the source of annotation (column "annotation_cellchat") that are performed by CellChat. In particular, the user has the option to exclude unwanted pathways from a drop-down menu and deselect unwanted annotations among "secreted signaling", "ECM-receptor", and "cell-cell contact".

CellPhoneDB v2

Users that chose to run CPDB to obtain predicted CCI data will have the option to refine int-pairs by excluding annotation sources used by CPDB to collect int-pairs, which are available in column "annotation_strategy". These include multiple sources such as, for example, "curated", "IMEx", "IntAct", "I2D", etc.

ICELLNET

Due to the fact that no additional annotation is provided by ICELLNET, no filtering options are available here.

SingleCellSignalR

As SCSR provides information regarding the specificity of an int-pair (see Supplementary Note 1), the user has the option to restrict the analysis to only specific interactions.

Supplementary Note 3: Comparison with CellChat

Many tools that perform cell-cell communication inference are available to the scientific community[5]. Comparing differences and similarities between InterCellar and existing applications might help the reader place InterCellar in this landscape. Among the tools that provide functionalities for downstream analysis, we chose CellChat, whose workflow appears to have many commonalities with InterCellar. Even though a Shiny implementation of CellChat exists, we decided to consider the R package which, although requiring programming skills, provides visualization and analysis options comparable to InterCellar. The CellChat Shiny app (https://github.com/sqjin/CellChatShiny), instead, only provides a limited set of analysis options, restricted to an initial choice of signaling pathways of interest. To run CellChat, we followed the tutorial available at https://htmlpreview.github.io/?https://github.com/sqjin/CellChat/blob/master/tutorial/CellChat-vignette.html.

The main focus of our comparison are visualization options provided by each tool and, specifically, how these can improve the interpretation of complex results in cell-cell communication analysis.

As a first overview of the communication patterns, the number of interactions occurring between each pair of clusters is generally considered. In InterCellar, this can be achieved in the cluster-verse (Figure 2a): when comparing the network visualization generated by InterCellar with the circle plot output of CellChat (Supplementary Figure 6a), we can appreciate how, even though an analogous output is produced, InterCellar's added value is an interactive network that can be dynamically remodeled by the user with a simple drag-and-drop. Moreover, clicking on a cell cluster will highlight all the connected edges, facilitating the inspection of large networks (these features are visible in the video tutorial at https://youtu.be/X5gUqzps4E4). Finally, both InterCellar and CellChat provide the option to show either the total or the weighted number of interactions.

A second step in the analysis could focus on the occurrence of int-pairs of interest, shown typically in a dot (or bubble) plot. Thus, we compare here InterCellar's dot plot available in the gene-verse (Figure 2b), with CellChat's bubble plot (Supplementary Figure 6b), both generated by considering all int-pairs annotated by CellChat as belonging to the "TGFb" signaling pathway. InterCellar and CellChat perform comparably well and offer customization options to the user (e.g., choice of color scheme, selection of cell clusters).

Lastly, we investigate visualization options based on annotated pathways, thus comparing InterCellar's sunburst plot (available in the function-verse, Figure 2c) with the closest kind of

visualization available in CellChat (Supplementary Figure 6c). InterCellar's sunburst plot is generated by selecting the functional term "tgf-beta signaling pathway" (annotated from KEGG and Panther) and, upon closer inspection of the enriched int-pairs, includes CellChat pathways corresponding to "TGFb", "BMP", "GDF" and "ACTIVIN". With regards to interpretation, the sunburst plot offers the advantage of condensing many sources of information in one place. Thus, cluster-pairs enriched by the selected functional term are visible and organized in such a way as to convey information regarding cluster importance. Detailed information on the (total/weighted) number of interactions as well as int-pairs occurring in each cluster-pair are available upon mouse hovering on the sunburst sections. As for the CellChat chord plot, even though the overall contribution of each cell cluster is clearly visible, the user must consider a second complementary visualization to discern enriched int-pairs (Supplementary Figure 6d). Moreover, the lack of a dynamic output for the CellChat chord plots can hinder an effective interpretation of the results in overcrowded plots.

In the end, we extend our comparative analysis on the concept of functional similarity, which is considered by both InterCellar and CellChat, albeit from two different points of view. Specifically, InterCellar calculates functional similarity among int-pairs, in contrast to the strategy adopted by CellChat where groups of pathways are defined to be functionally similar. Considering two different "similarity objects", thus leads to parallel and rather complementary analyses. This can be specifically appreciated by comparing InterCellar's UMAP of int-pairs clustered by functional similarity (Figure 3a), and CellChat's embedding of pathways clustered by functional similarity (Supplementary Figure 7). While InterCellar defines modules of int-pairs as groups of int-pairs that share a similar functional profile (Supplementary Figure 2), CellChat groups functional pathways by a shared pattern of sender and receiver clusters.

Supplementary Figures

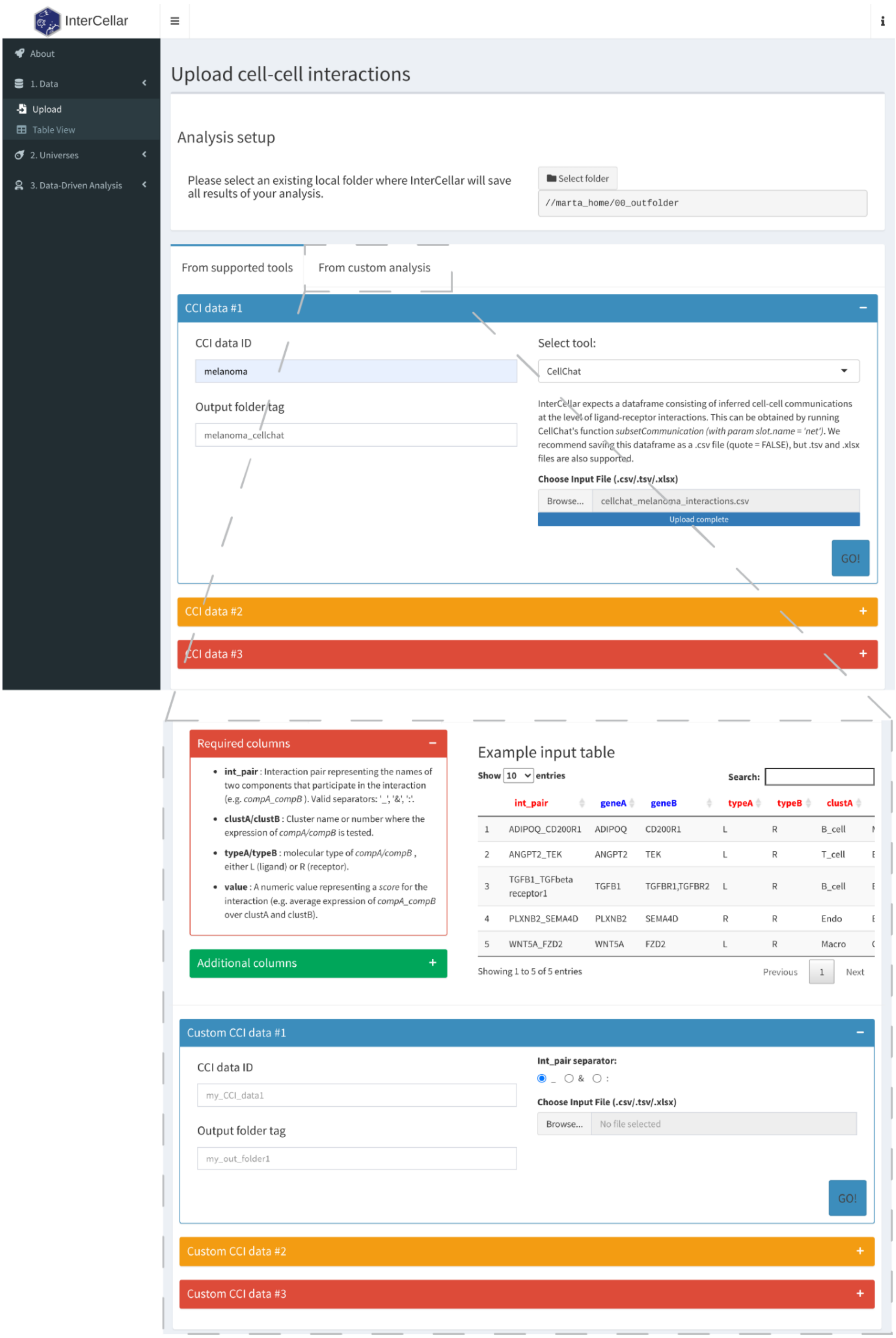
InterCellar
About
1. Data
Upload
Table View
2. Universes
3. Data-Driven Analysis
Upload cell-cell interactions
Analysis setup
Please select an existing local folder where InterCellar will save all results of your analysis.
Select folder
//marta_home/00_outfolder
From supported tools
From custom analysis
CCI data #1
CCI data ID
melanoma
Output folder tag
melanoma_cellchat
Select tool:
CellChat
InterCellar expects a dataframe consisting of inferred cell-cell communications at the level of ligand-receptor interactions. This can be obtained by running CellChat's function subsetCommunication (with param slot.name = 'net'). We recommend saving this dataframe as a .csv file (quote = FALSE), but .tsv and .xlsx files are also supported.
Choose Input File (.csv/.tsv/.xlsx)
Browse...
cellchat_melanoma_interactions.csv
Upload complete
GO!
CCI data #2
CCI data #3
Required columns
int_pair : Interaction pair representing the names of two components that participate in the interaction (e.g. compA_compB). Valid separators: '_', '&', ':'.
clustA/clustB : Cluster name or number where the expression of compA/compB is tested.
typeA/typeB : molecular type of compA/compB , either L (ligand) or R (receptor).
value : A numeric value representing a score for the interaction (e.g. average expression of compA_compB over clustA and clustB).
Additional columns
Example input table
Show 10 entries
Search:
int_pair geneA geneB typeA typeB clustA
1 ADIPOQ_CD200R1 ADIPOQ CD200R1 L R B_cell
2 ANGPT2_TEK ANGPT2 TEK L R T_cell
3 TGFB1_TGFbeta receptor1 TGFB1 TGFBR1,TGFBR2 L R B_cell
4 PLXNB2_SEMA4D PLXNB2 SEMA4D R R Endo
5 WNT5A_FZD2 WNT5A FZD2 L R Macro
Showing 1 to 5 of 5 entries
Previous 1 Next
Custom CCI data #1
CCI data ID
my_CCI_data1
Output folder tag
my_out_folder1
Int_pair separator:
_ & :
Choose Input File (.csv/.tsv/.xlsx)
Browse...
No file selected
GO!
Custom CCI data #2
Custom CCI data #3

Supplementary Figure 1. Screenshot of InterCellar's data upload module. The top panel (Analysis setup) allows the selection of an output folder in which InterCellar will save all downloaded figures and tables in an intuitive folder structure. The lower panel (from supported tools) provides functionalities to upload up to three CCI data generated by InterCellar's supported input methods. Here, we upload CellChat-predicted CCI data for the melanoma dataset[6] by specifying an ID, an output folder tag, and the file containing predicted CCI. The bottom inset shows the panel "from custom analysis". Here, a CCI input table is displayed as an example, alongside the description of required and additional columns. Once again, up to three custom CCI data can be uploaded by the user.

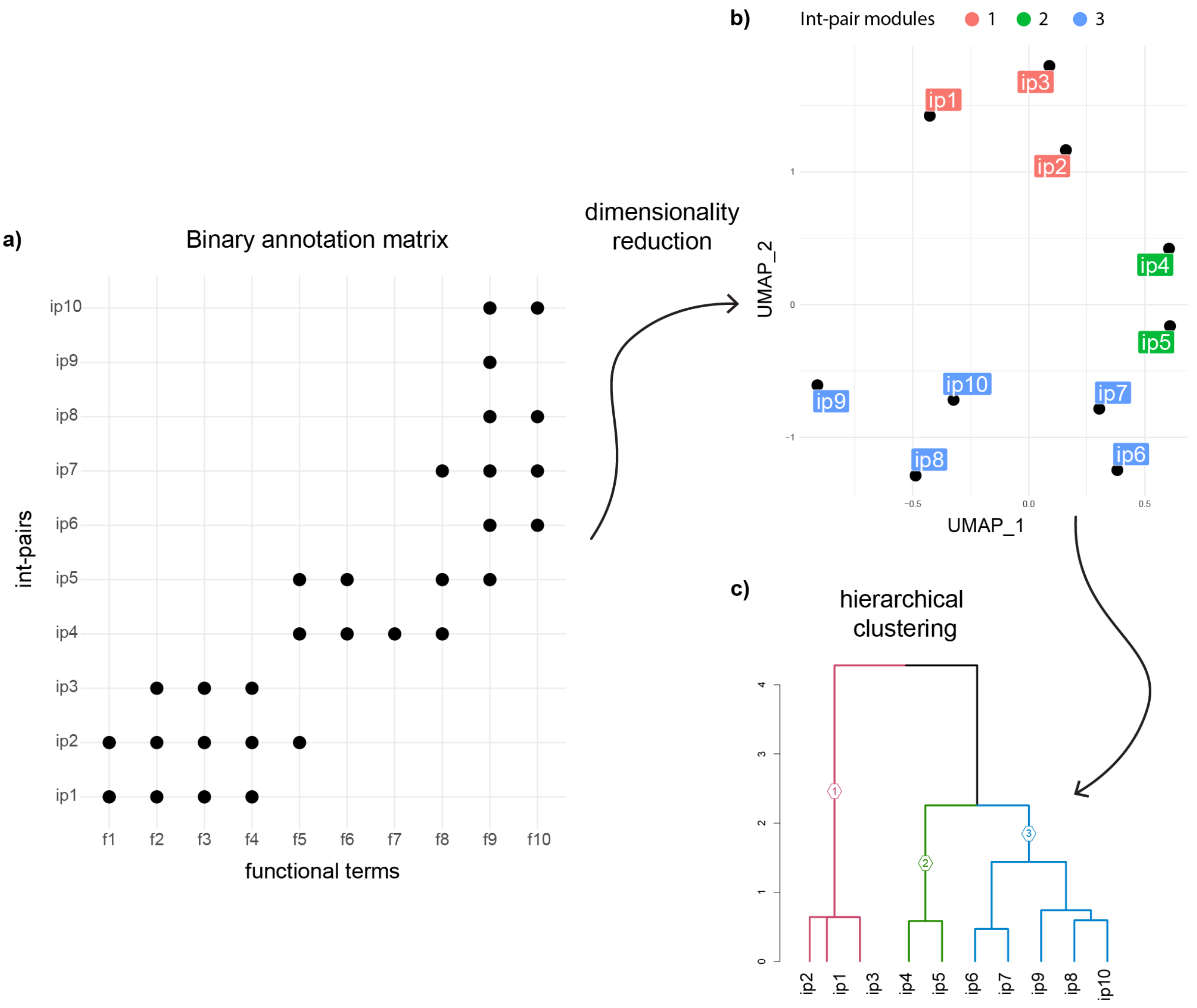

Supplementary Figure 2. InterCellar's definition of int-pair modules based on functional similarity. A representative binary (boolean domain {0,1}) annotation matrix is shown in **a)**, where black dots indicate "1s" and thus an existing annotation of the functional

term “f-” to an int-pair “ip-”. InterCellar uses this binary matrix as input to a dimensionality reduction method (UMAP in **b)**, using cosine similarity as metric to compute distances between data points), which provides a 2-dimensional embedding of int-pairs reflecting the functional similarity. Using the UMAP coordinates, hierarchical clustering is computed to define modules of int-pairs that share similar functional profiles (with euclidean distance and ward.D2 clustering algorithm), as shown in **c)**. Colors represent the module identity in both **b)**, UMAP and **c)**, dendrogram.

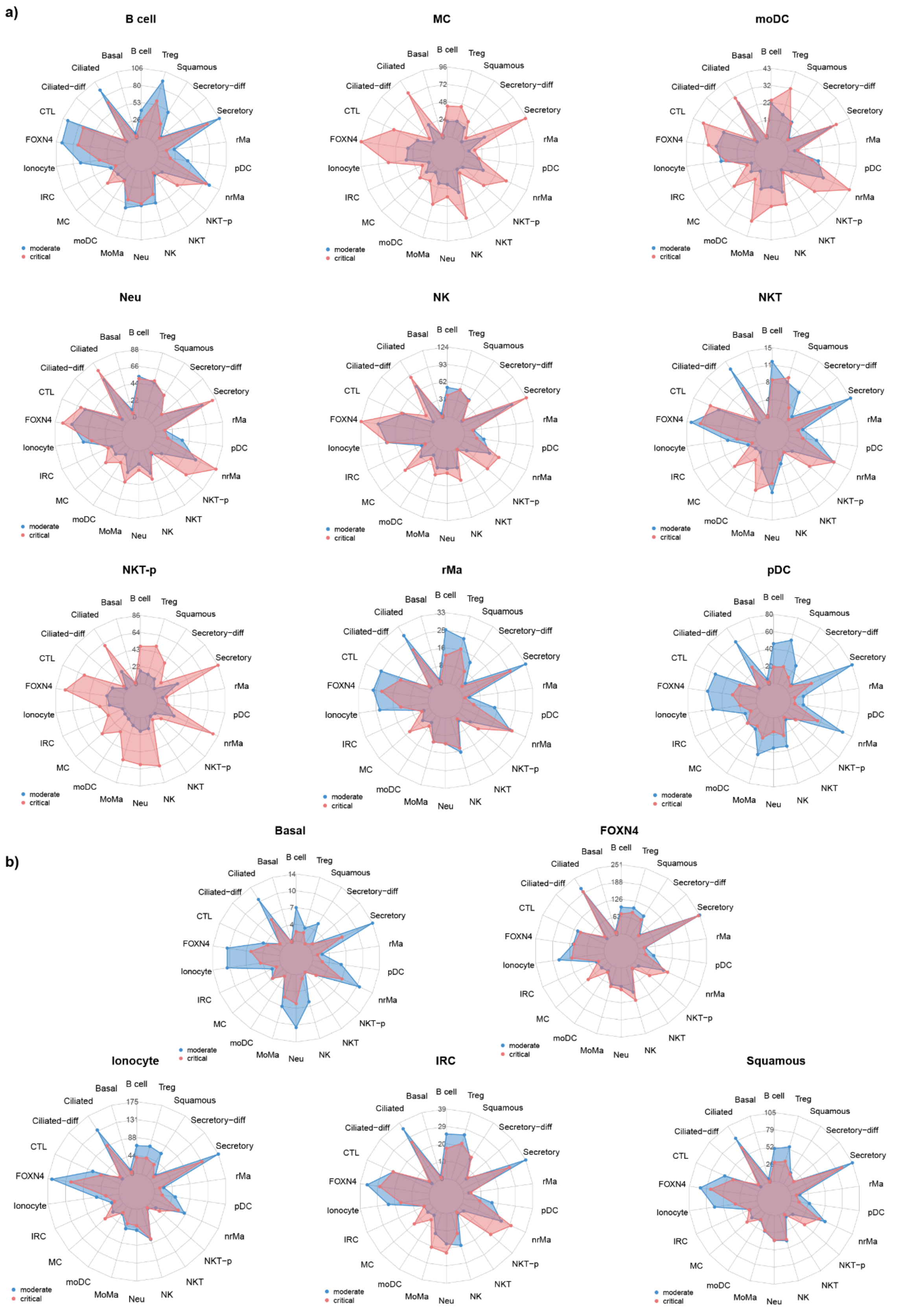

Supplementary Figure 3. InterCellar generates comprehensive insights on the number of interactions per cell cluster. Relative differences in the number of interactions are

shown using radar plots, in the comparison between COVID-19 moderate and critical cases[7]. Each plot displays the number of interactions between a specific cell type and all other cell types participating in the communication. All interaction flows are considered. A subset of cell types belonging to the immune group is reported in **a)**, while a subset of epithelial-related cell types is shown in **b)**. Radar plots are generated automatically in InterCellar's multiple conditions section.

Cell types are abbreviated as: -diff - differentiating; CTL - cytotoxic T cell; FOXN4 - FOXN4+ epithelial cells; IRC - IFNG responsive cell; MC - mast cell; moDC - monocyte-derived dendritic cell; MoMa - monocyte-derived macrophage; Neu - neutrophil; NK - natural killer cell; NKT - NK T cell; NKT-p - proliferating NKT; nrMa - non-resident macrophage; pDC - plasmacytoid dendritic cell; rMa - resident macrophage; Treg - regulatory T cell.

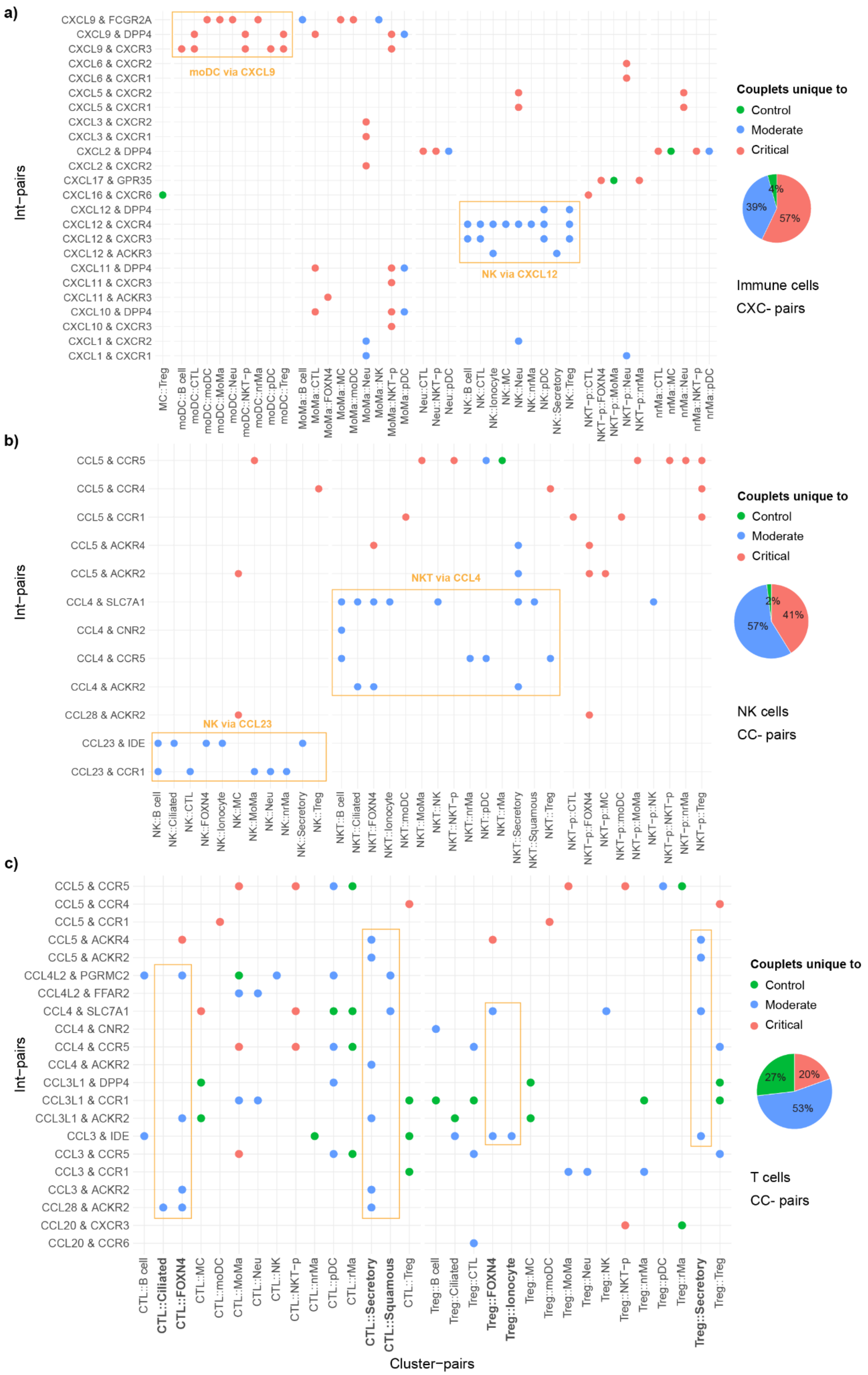
a)
CXCL9 & FCGR2A
CXCL9 & DPP4
CXCL9 & CXCR3
CXCL6 & CXCR2
CXCL6 & CXCR1
CXCL5 & CXCR2
CXCL5 & CXCR1
CXCL3 & CXCR2
CXCL3 & CXCR1
CXCL2 & DPP4
CXCL2 & CXCR2
CXCL17 & GPR35
CXCL16 & CXCR6
CXCL12 & DPP4
CXCL12 & CXCR4
CXCL12 & CXCR3
CXCL12 & ACKR3
CXCL11 & DPP4
CXCL11 & CXCR3
CXCL11 & ACKR3
CXCL10 & DPP4
CXCL10 & CXCR3
CXCL1 & CXCR2
CXCL1 & CXCR1
Int−pairs
moDC via CXCL9
NK via CXCL12
MC::Treg
moDC::B cell
moDC::CTL
moDC::moDC
moDC::MoMa
moDC::Neu
moDC::NKT−p
moDC::nrMa
moDC::pDC
moDC::Treg
MoMa::B cell
MoMa::CTL
MoMa::FOXN4
MoMa::MC
MoMa::moDC
MoMa::Neu
MoMa::NK
MoMa::NKT−p
MoMa::pDC
Neu::CTL
Neu::NKT−p
Neu::pDC
NK::B cell
NK::CTL
NK::Ionocyte
NK::MC
NK::Neu
NK::nrMa
NK::pDC
NK::Secretory
NK::Treg
NKT−p::CTL
NKT−p::FOXN4
NKT−p::MoMa
NKT−p::Neu
NKT−p::nrMa
nrMa::CTL
nrMa::MC
nrMa::Neu
nrMa::NKT−p
nrMa::pDC
Couplets unique to
Control
Moderate
Critical
4%
39%
57%
Immune cells
CXC- pairs
b)
CCL5 & CCR5
CCL5 & CCR4
CCL5 & CCR1
CCL5 & ACKR4
CCL5 & ACKR2
CCL4 & SLC7A1
CCL4 & CNR2
CCL4 & CCR5
CCL4 & ACKR2
CCL28 & ACKR2
CCL23 & IDE
CCL23 & CCR1
Int−pairs
NKT via CCL4
NK via CCL23
NK::B cell
NK::Ciliated
NK::CTL
NK::FOXN4
NK::Ionocyte
NK::MC
NK::MoMa
NK::Neu
NK::nrMa
NK::Secretory
NK::Treg
NKT::B cell
NKT::Ciliated
NKT::FOXN4
NKT::Ionocyte
NKT::moDC
NKT::MoMa
NKT::NK
NKT::NKT−p
NKT::nrMa
NKT::pDC
NKT::rMa
NKT::Secretory
NKT::Squamous
NKT::Treg
NKT−p::CTL
NKT−p::FOXN4
NKT−p::MC
NKT−p::moDC
NKT−p::MoMa
NKT−p::NK
NKT−p::NKT−p
NKT−p::nrMa
NKT−p::Treg
Couplets unique to
Control
Moderate
Critical
2%
41%
57%
NK cells
CC- pairs
c)
CCL5 & CCR5
CCL5 & CCR4
CCL5 & CCR1
CCL5 & ACKR4
CCL5 & ACKR2
CCL4L2 & PGRMC2
CCL4L2 & FFAR2
CCL4 & SLC7A1
CCL4 & CNR2
CCL4 & CCR5
CCL4 & ACKR2
CCL3L1 & DPP4
CCL3L1 & CCR1
CCL3L1 & ACKR2
CCL3 & IDE
CCL3 & CCR5
CCL3 & CCR1
CCL3 & ACKR2
CCL28 & ACKR2
CCL20 & CXCR3
CCL20 & CCR6
Int−pairs
CTL::B cell
CTL::Ciliated
CTL::FOXN4
CTL::MC
CTL::moDC
CTL::MoMa
CTL::Neu
CTL::NK
CTL::NKT−p
CTL::nrMa
CTL::pDC
CTL::rMa
CTL::Secretory
CTL::Squamous
CTL::Treg
Treg::B cell
Treg::Ciliated
Treg::CTL
Treg::FOXN4
Treg::Ionocyte
Treg::MC
Treg::moDC
Treg::MoMa
Treg::Neu
Treg::NK
Treg::NKT−p
Treg::nrMa
Treg::pDC
Treg::rMa
Treg::Secretory
Treg::Treg
Cluster−pairs
Couplets unique to
Control
Moderate
Critical
27%
20%
53%
T cells
CC- pairs

Supplementary Figure 4. InterCellar allows in-depth analysis of interaction pairs and their enriched cell clusters. a) Int-pairs belonging to the *CXC*-chemokine subfamily are evaluated in immune cell clusters. The dot plot represents only unique occurrences of int-pair/cluster-pair couplets, for each phenotype (control, moderate, critical). The overall contribution of each phenotype is summarized in a pie chart. **b)** Int-pairs belonging to the *CC*-chemokine subfamily are evaluated in NK, NKT, and NKT-p cell clusters. As in **a)**, only unique couplets are represented in the dot plot and overall contributions by phenotype are summarized in a pie chart. **c)** As in **b)**, but focusing on T cells (CTL and Tregs). Both dot plots and pie charts are generated in InterCellar's multiple conditions section.

	**InterCellar**	**CCCExplorer**	**pyMINEr**	**CellChat Explorer**	**Cellinker**	**talkIr**
Programming skills	✗	✗	✗	✗	✗	✗
Interactive analysis	✓	✓	✗	✗	✗	✗
Local or remote	local	local	local	remote	remote	remote
Data type	scRNA-seq	bulk RNA-seq	scRNA-seq	scRNA-seq	scRNA-seq	scRNA-seq
Customization/filtering	✓	✓	✗	✗	✗	✗
Visualization	✓	✓	✗	✓	✓	✓
Cluster-based	✓	✗		✓	✗	✓
Gene-based	✓	✓		✓	✓	✗
Function-based	✓	✓		✓	✗	✗
Download	✓	✓	✓	✗	✗	✓
Figures	✓	✓	✗			✗
Tables	✓	✓	✓			✓

Supplementary Figure 5. Feature comparison between InterCellar and other related tools. Seven main categories evaluate InterCellar's functionalities against other open-source software allowing the analysis of cell-cell communication. For this comparison, only tools that do not require programming skills were considered. Visualization options are divided into three sub-categories to account for specific features implemented with regards to cell clusters, genes, and functional pathways. Automatic download of figures and tables is considered separately.

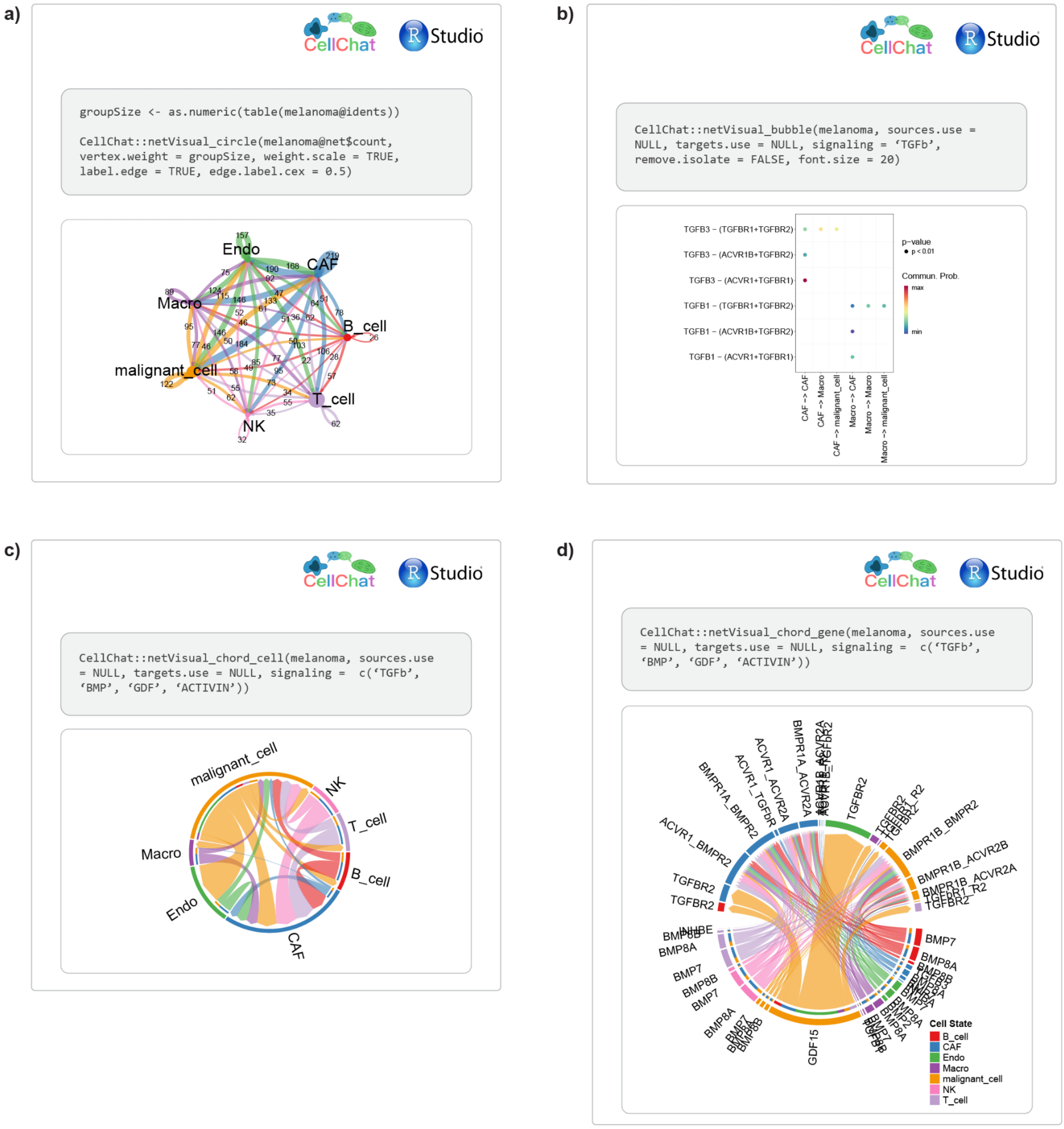

Supplementary Figure 6. Visualization options provided by the CellChat R package for data exploration. a) Network-like visualization generated by CellChat is presented alongside the R code used to obtain the output. Edges show the total number of interactions occurring between two cell clusters. **b)** CellChat's bubble plot is shown alongside the R code necessary to generate it. All interaction pairs annotated to the "TGFb" signaling pathway are selected. **c)** CellChat's chord plot and the relative R code, for the selected pathways ("TGFb", "BMP", "GDF", and "ACTIVIN"). Cell clusters are represented in the outer circle, with colored links indicating the presence of interactions between the connected clusters. **d)** R code and visualization of CellChat's chord plot relative to the interaction pairs enriched in the selected pathways ("TGFb", "BMP", "GDF", and "ACTIVIN"). Ligands and receptors are

shown in the outer circle, with colored links representing the interactions between two clusters (color-coded).

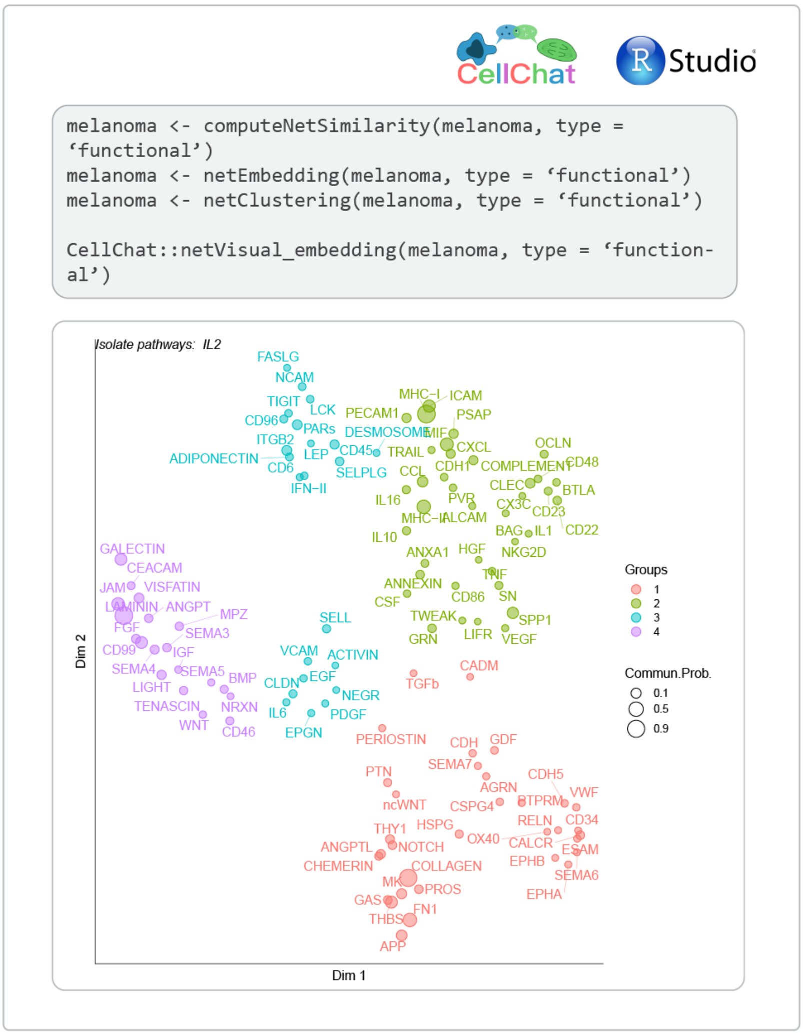

Supplementary Figure 7. CellChat embedding of pathways based on functional similarity. UMAP of CellChat-annotated pathways shows the results of the functional similarity analysis, with R code available in the gray panel. Each dot represents a pathway, color-coded by the group identity. Pathways that are found to be functionally similar share a common pattern of sender and receiver clusters and can be interpreted as signaling pathways that exhibit similar and/or redundant roles.

Supplementary Tables

	Implementation	Standalone app	App web address	Data type	DOI	Source code

**CCCExplorer**	Java	YES	N/A	bulk RNA-seq	10.1016/j.celrep.2015.01.040	https://github.com/methodistsmab/CCCExplorer
**CellChat**	R	YES	http://www.cellchat.org/	scRNA-seq	10.1038/s41467-021-21246-9	https://github.com/sqjin/CellChat
**Cellinker**	HTML/PHP + mySQL	YES	http://www.rna-society.org/cellinker/	scRNA-seq	10.1093/bioinformatics/btab036	N/A
**CellPhoneDB**	Python	disabled upload	https://www.cellphonedb.org/explore-sc-rna-seq	scRNA-seq	10.1038/s41596-020-0292-x	https://github.com/Teichlab/cellphonedb
**CellTalker**	R	NO	N/A	scRNA-seq	10.1016/j.immuni.2019.11.014	https://github.com/arc85/celltalker
**COMUNET**	R	NO	N/A	scRNA-seq	10.1093/bioinformatics/btaa482	https://github.com/ScialdoneLab/COMUNET
**Connectome**	R	NO	N/A	scRNA-seq	10.1101/2021.01.21.427529	https://github.com/msraredon/Connectome
**CrossTalkeR**	R	NO	N/A	scRNA-seq	10.1101/2021.01.20.427390	https://github.com/CostaLab/CrossTalkeR
**ICELLNET**	R	NO	N/A	scRNA-seq	10.1101/2020.03.05.976878	https://github.com/soumelis-lab/ICELLNET
**iTALK**	R	NO	N/A	scRNA-seq	10.1101/507871	https://github.com/Coolgenome/iTALK
**NATMI**	Python	NO	N/A	bulk and scRNA-seq	10.1038/s41467-020-18873-z	https://github.com/forrest-lab/NATMI
**NicheNet**	R	NO	N/A	bulk and scRNA-seq	10.1038/s41592-019-0667-5	https://github.com/saeyslab/nichenetr
**pyMINEr**	Python	YES	https://www.sciencescott.com/pyminer	scRNA-seq	10.1016/j.celrep.2019.01.063	https://www.sciencescott.com/pyminer

**scTensor**	R	NO	N/A	scRNA-seq	10.1101/566182	https://github.com/rikenbit/scTensor
**SingleCellSignalR**	R	NO	N/A	scRNA-seq	10.1093/nar/gkaa183	https://github.com/SCA-IRCM/SingleCellSignalR_v1
**SoptSC**	R/MATLAB	NO	N/A	scRNA-seq	10.1093/nar/gkz204	https://mkarikom.github.io/RSoptSC/
**SpaOTsc**	Python	NO	N/A	scRNA-seq	10.1038/s41467-020-15968-5	https://github.com/zcang/SpaOTsc
**talklr**	R	YES	https://yuliangwang.shinyapps.io/talklr/	scRNA-seq	10.1101/2020.02.01.930602	https://github.com/yuliangwang/talklr

Supplementary Table 1. Collection of methods to predict and analyze cell-cell communication. The table contains 18 methods retrieved as of April 2021. All methods concern cell-cell communication prediction and/or analysis. The following information was collected: (a) programming language used for implementation; (b) whether the method is available as a standalone application (thus, not requiring programming skills to conduct the analysis); (c) corresponding web address of the application; (d) data type required for the prediction of cell-cell interactions; (e) DOI of the corresponding manuscript; and (f) web address of the source code. Five methods that do not require programming skills (shown with colored background) were compared to InterCellar.

**source**	**target**	**ligand**	**receptor**	**prob**	**pval**	**interaction_name**	**interaction_name_2**	**pathway_name**	**annotation**	**evidence**
Macro	CAF	*TGFB1*	*TGFbR1_R2*	0.000210484	0	TGFB1_TGFBR1_TGFBR2	TGFB1 - (TGFBR1+TGFBR2)	TGFb	Secreted Signaling	KEGG: hsa04350

Macro	Macro	*TGFB1*	*TGFbR1_R2*	0.000694483	0	TGFB1_TGFBR1_TGFBR2	TGFB1 - (TGFBR1+TGFBR2)	TGFb	Secreted Signaling	KEGG: hsa04350
Macro	malignant_cell	*TGFB1*	*TGFbR1_R2*	0.000513576	0	TGFB1_TGFBR1_TGFBR2	TGFB1 - (TGFBR1+TGFBR2)	TGFb	Secreted Signaling	KEGG: hsa04350
CAF	CAF	*TGFB3*	*TGFbR1_R2*	0.000934753	0	TGFB3_TGFBR1_TGFBR2	TGFB3 - (TGFBR1+TGFBR2)	TGFb	Secreted Signaling	KEGG: hsa04350
CAF	Macro	*TGFB3*	*TGFbR1_R2*	0.003093581	0	TGFB3_TGFBR1_TGFBR2	TGFB3 - (TGFBR1+TGFBR2)	TGFb	Secreted Signaling	KEGG: hsa04350

Supplementary Table 2. Example of CellChat CCI data as input to InterCellar. Example of CCI data as generated by CellChat, automatically parsed by InterCellar as input data.

**id_cp_interaction**	**interacting_pair**	**partner_a**	**partner_b**	**gene_a**	**gene_b**	**secreted**	**receptor_a**	**receptor_b**	**...**
CPI-SC05F111D21	COL27A1_a11b1 complex	simple:Q8IZC6	complex:a11b1 complex	COL27A1		TRUE	FALSE	FALSE	...
CPI-SS016EE0084	PDCD1_CD274	simple:Q15116	simple:Q9NZQ7	PDCD1	CD274	FALSE	TRUE	TRUE	...
CPI-SS	KIR2D	simple:	simple:	KIR2D	CXCL9	TRUE	TRUE	FALSE	...

<table>
<tr><td>0D26F639E</td><td>L3_CXCL9</td><td>P43628</td><td>Q07325</td><td>L3</td><td></td><td></td><td></td><td></td><td></td></tr>
<tr><td>CPI-CS0AC5142FA</td><td>IL1 receptor inhibitor_IL1A</td><td>complex:IL1 receptor inhibitor</td><td>simple:P01583</td><td></td><td>IL1A</td><td>TRUE</td><td>TRUE</td><td>FALSE</td><td>...</td></tr>
<tr><td>...</td><td>...</td><td>...</td><td>...</td><td>...</td><td>...</td><td>...</td><td>...</td><td>...</td><td>...</td></tr>
<tr><td>...</td><td>annotation_strategy</td><td>is_integrin</td><td>rank</td><td>B_cell|B_cell</td><td>B_cell|CAF</td><td>B_cell|Endo</td><td>B_cell|Macro</td><td>B_cell|NK</td><td>...</td></tr>
<tr><td>...</td><td>curated</td><td>TRUE</td><td>0.02</td><td></td><td></td><td></td><td></td><td>0.034</td><td>...</td></tr>
<tr><td>...</td><td>curated</td><td>FALSE</td><td>0.02</td><td></td><td>0.816</td><td></td><td></td><td></td><td>...</td></tr>
<tr><td>...</td><td>IMEx,IntAct</td><td>FALSE</td><td>0.02</td><td></td><td></td><td></td><td></td><td></td><td>...</td></tr>
<tr><td>...</td><td>curated</td><td>FALSE</td><td>0.02</td><td></td><td></td><td></td><td>1.055</td><td></td><td>...</td></tr>
</table>

Supplementary Table 3. Example of CellPhoneDB v2 "significant_means" table as input to InterCellar. Four example rows are taken from the significant_means.txt file saved by CPDB statistical analysis. The table is split after the ninth column for easier interpretation. Not all columns are shown, as they are comprised of all possible cluster-pairs in the dataset.

	**CAF**	**Macro**	**T_cell**	**B_cell**	**Endo**	**NK**
AMH / BMPR1A + AMHR2	0	0	1.179866138	0.637237869	0	0
AMH / ACVR1 + AMHR2	0	3.85242671	1.217964923	0.451851926	0	0
AREG / EGFR	2.374966797	0.00930926	0.017255723	0.042389732	0.171259444	0.163602413
BMP10 /	3.7620708	2.1915530	0.9590051	0.6822325	5.4375626	1.0956195

ACVRL1 + ACVR2A	48	94	28		33	23
BMP10 / ACVRL1 + ACVR2B	1.924009552	1.2200742	0.922325368	0.903325695	4.462582679	0

Supplementary Table 4. Example of ICELLNET CCI data as input to InterCellar. Example of CCI data as generated by ICELLNET, when considering malignant cells as central cell and outgoing communication. Row names represent the interaction pairs while column names represent the cell clusters considered in the analysis, communicating with malignant cells.

**CAF**	**malignant_cell**	**interaction.type**	**LRscore**
TIMP1	*CD63*	autocrine|paracrine	0.943270962296891
VIM	*CD44*	autocrine|paracrine	0.930737459594635
COL1A2	*CD44*	autocrine|paracrine	0.928081880647428

Supplementary Table 5. Example of SingleCellSignalR text file as input to InterCellar. SingleCellSignalR generates a text file for each cluster-pair and interaction type (autocrine/paracrine) considered and saves it in the “cell-signaling” folder, which is expected as input to InterCellar. Here, the interactions between CAF and malignant cells are computed.

Supplementary References

1. Jin, S. *et al.* Inference and analysis of cell-cell communication using CellChat. *Nat. Commun.* **12**, 1088 (2021).
2. Efremova, M., Vento-Tormo, M., Teichmann, S. A. & Vento-Tormo, R. CellPhoneDB: inferring cell–cell communication from combined expression of multi-subunit

ligand–receptor complexes. *Nat. Protoc.* **15**, 1484–1506 (2020).

3. Cabello-Aguilar, S. *et al.* SingleCellSignalR: inference of intercellular networks from single-cell transcriptomics. *Nucleic Acids Res.* **48**, e55 (2020).
4. Noël, F. *et al.* Dissection of intercellular communication using the transcriptome-based framework ICELLNET. *Nat. Commun.* **12**, 1089 (2021).
5. Armingol, E., Officer, A., Harismendy, O. & Lewis, N. E. Deciphering cell–cell interactions and communication from gene expression. *Nat. Rev. Genet.* **22**, 71–88 (2020).
6. Tirosh, I. *et al.* Dissecting the multicellular ecosystem of metastatic melanoma by single-cell RNA-seq. *Science* **352**, 189–196 (2016).
7. Chua, R. L. *et al.* COVID-19 severity correlates with airway epithelium-immune cell interactions identified by single-cell analysis. *Nat. Biotechnol.* **38**, 970–979 (2020).
