# Supplementary material for: InterCellar enables interactive analysis and exploration of cell−cell communication in single-cell transcriptomic data
Source: Commun Biol. 2022 Jan 11;5:21. doi: 10.1038/s42003-021-02986-2 (PMC8752611; doi:10.1038/s42003-021-02986-2)
Supplement: Supplementary file 3 — Description of Additional Supplementary Files [file 42003_2021_2986_MOESM3_ESM.pdf]

Description of Additional Supplementary Files

File name: Supplementary Data 1

Description: The tables contain total number of interactions per cluster in the COVID-19 critical and moderate cases, used to generate Figure 4a. Paracrine and autocrine interactions are split in two columns.
