# Supplementary material for: InterCellar enables interactive analysis and exploration of cell−cell communication in single-cell transcriptomic data
Source: Commun Biol. 2022 Jan 11;5:21. doi: 10.1038/s42003-021-02986-2 (PMC8752611; doi:10.1038/s42003-021-02986-2)
Supplement: Supplementary file 5 — Reporting Summary [file 42003_2021_2986_MOESM5_ESM.pdf]

nature research

Corresponding author(s): Marta Interlandi

Last updated by author(s): Nov 29, 2021

Reporting Summary

Nature Research wishes to improve the reproducibility of the work that we publish. This form provides structure for consistency and transparency in reporting. For further information on Nature Research policies, see our Editorial Policies and the Editorial Policy Checklist.

Statistics

For all statistical analyses, confirm that the following items are present in the figure legend, table legend, main text, or Methods section.

n/a	Confirmed	
☐	☒	The exact sample size (*n*) for each experimental group/condition, given as a discrete number and unit of measurement
☐	☒	A statement on whether measurements were taken from distinct samples or whether the same sample was measured repeatedly
☐	☒	The statistical test(s) used AND whether they are one- or two-sided<br>*Only common tests should be described solely by name; describe more complex techniques in the Methods section.*
☒	☐	A description of all covariates tested
☒	☐	A description of any assumptions or corrections, such as tests of normality and adjustment for multiple comparisons
☐	☒	A full description of the statistical parameters including central tendency (e.g. means) or other basic estimates (e.g. regression coefficient) AND variation (e.g. standard deviation) or associated estimates of uncertainty (e.g. confidence intervals)
☐	☒	For null hypothesis testing, the test statistic (e.g. *F*, *t*, *r*) with confidence intervals, effect sizes, degrees of freedom and *P* value noted<br>*Give P values as exact values whenever suitable.*
☒	☐	For Bayesian analysis, information on the choice of priors and Markov chain Monte Carlo settings
☒	☐	For hierarchical and complex designs, identification of the appropriate level for tests and full reporting of outcomes
☒	☐	Estimates of effect sizes (e.g. Cohen's *d*, Pearson's *r*), indicating how they were calculated

Our web collection on statistics for biologists contains articles on many of the points above.

Software and code

Policy information about availability of computer code

Data collection	All data analyzed within this manuscript are publicly available.<br>For the COVID-19 dataset, CellPhoneDB v2.0.0 was run with default parameters to obtain cell-cell interaction data as input to InterCellar.<br>For the melanoma dataset, CellChat v1.1.2 was run to obtain cell-cell interaction data as input to InterCellar.<br>InterCellar's input datasets used in this study are available at https://github.com/martaint/InterCellar-reproducibility, as well as code to generate them.
Data analysis	We used the InterCellar R/Bioconductor package (v2.0.0) to analyze cell-cell communication data (https://bioconductor.org/packages/InterCellar/)<br>Source code for the package is available at https://github.com/martaint/InterCellar.<br>Tutorials on how to reproduce the analysis presented in the manuscript are available at https://github.com/martaint/InterCellar-reproducibility.

For manuscripts utilizing custom algorithms or software that are central to the research but not yet described in published literature, software must be made available to editors and reviewers. We strongly encourage code deposition in a community repository (e.g. GitHub). See the Nature Research guidelines for submitting code & software for further information.

Data

Policy information about availability of data

All manuscripts must include a data availability statement. This statement should provide the following information, where applicable:

- Accession codes, unique identifiers, or web links for publicly available datasets
- A list of figures that have associated raw data
- A description of any restrictions on data availability

COVID-19 datasets were retrieved from FigShare at https://doi.org/10.6084/m9.figshare.12436517.v2 (data object named "covid_nbt_main.rds").
For the melanoma dataset, preprocessed data were downloaded from GEO with Accession Number GSE72056.

Field-specific reporting

Please select the one below that is the best fit for your research. If you are not sure, read the appropriate sections before making your selection.

☒ Life sciences ☐ Behavioural & social sciences ☐ Ecological, evolutionary & environmental sciences

For a reference copy of the document with all sections, see nature.com/documents/nr-reporting-summary-flat.pdf

Life sciences study design

All studies must disclose on these points even when the disclosure is negative.

Sample size	No sample size calculation was performed. All data used in this manuscript were taken from public resources and used to demonstrate the functionalities of InterCellar. The COVID-19 dataset comprises 3 conditions (control, COVID19-moderate and COVID-19 critical) that were used alone, or in comparison. The melanoma dataset has only one condition.
Data exclusions	COVID-19: we removed two cell clusters whose label assignment was poorly defined, namely "unknown epithelial" and "outlier epithelial", corresponding to ~1.5% of the total number of cells. Moreover, moderate and critical datasets were randomly subsampled to 10,000 cells each (without losing any cell label), while for the control dataset we retained all cells, corresponding to a total of 2,966 cells.<br>MELANOMA: We removed ~11% of these cells, due to a missing or unknown cell type label, retaining 4,097 final cells.
Replication	All attempts at replication were successful and can be performed independently. The GitHub repository https://github.com/martaint/InterCellar-reproducibility contains data and tutorials to reproduce all findings reported in this manuscript.
Randomization	Not relevant for our study as we used conditions and cell types defined by the original authors of the published data.
Blinding	Blinding was not possible since we used published data.

Reporting for specific materials, systems and methods

We require information from authors about some types of materials, experimental systems and methods used in many studies. Here, indicate whether each material, system or method listed is relevant to your study. If you are not sure if a list item applies to your research, read the appropriate section before selecting a response.

Materials & experimental systems

n/a	Involved in the study	
☒	☐	Antibodies
☒	☐	Eukaryotic cell lines
☒	☐	Palaeontology and archaeology
☒	☐	Animals and other organisms
☒	☐	Human research participants
☒	☐	Clinical data
☒	☐	Dual use research of concern

Methods

n/a	Involved in the study	
☒	☐	ChIP-seq
☒	☐	Flow cytometry
☒	☐	MRI-based neuroimaging
